# Exercise During Chemotherapy for Cancer: A Systematic Review

**DOI:** 10.1002/jso.27845

**Published:** 2024-10-23

**Authors:** R. C. Walker, P. Pezeshki, S. Barman, S. Ngan, G. Whyte, J. Lagergren, J. Gossage, M. Kelly, C. Baker, W. Knight, M. A. West, A. R. Davies, Guy's & St Thomas' Oesophago‐gastric Research Group

**Affiliations:** ^1^ Guy's & St Thomas' Oesophago‐gastric Centre London UK; ^2^ School of Cancer Sciences, Faculty of Medicine University of Southampton Southampton UK; ^3^ School of Surgery & Cancer Imperial College London London UK; ^4^ School of Cancer & Pharmaceutical Sciences King's College London London UK; ^5^ Centre for Health and Human Performance London UK; ^6^ Karolinska Institutet Stockholm Sweden; ^7^ NIHR Southampton Biomedical Research Centre, Perioperative and Critical Care University Hospital Southampton NHS Foundation Trust Southampton UK

**Keywords:** chemotherapy, exercise prehabilitation, neoadjuvant therapy

## Abstract

Exercise prehabilitation may improve the tolerance and effectiveness of anticancer treatments such as chemotherapy. This systematic review assesses the impact of exercise on chemotherapy outcomes and identifies research priorities. Nineteen studies (1418 patients) were reviewed, including 11 randomised controlled trials and eight observational studies. Exercise led to improvements in body composition, fitness, strength and quality of life (QoL) across studies. Exercise can be safely and effectively delivered during chemotherapy. Limited standardisation and small sample sizes highlight the need for larger, better‐designed studies to optimise this low‐cost intervention.

## 1 Introduction

There is strong evidence that the systemic adverse effects of a cancer diagnosis and its treatment can be improved by exercise [1]. The American College of Sport Medicine (ACSM) has produced evidence‐based exercise prescriptions since 2010 [2, 3] to mitigate the physical and psychological side effects of cancer treatment based on improvements in anxiety, depressive symptoms, fatigue and physical function. Several studies have reported the positive effects of exercise on completion rates of chemotherapy, which may result in improved survival rates [4, 5]. Patients with reduced physical function following a cancer diagnosis, have poorer 10‐year survival [6].

Most existing research investigates the influence of exercise on morbidity, mortality, quality of life (QoL) and treatment side effects. While some studies also investigate the effect of exercise on tumour‐promoting inflammation, few studies have examined the effects on the tumour itself [7]. Although some studies have linked pain, cognitive impairment and fatigue to cell‐level biological effects such as increased pro‐inflammatory cytokines [8, 9], only a limited number of studies have looked at the effects of exercise on the progression of disease. It is widely acknowledged that the benefits of exercise may be broader and the mechanisms of these benefits are currently poorly understood.

This review seeks to appraise and summarise the evidence for the effect of exercise on the outcomes of patients undergoing chemotherapy and to define outstanding research questions.

## 2 Methods

### 2.1 Search

An electronic search of PubMed, Web of Science and the Cochrane Central Register of Controlled Trials (CENTRAL) was performed (February 2022), using the search term (‘exercise’[Mesh] OR ‘Exercise*’[tiab] OR ‘physical activity*’[tiab] OR ‘physical training’[tiab] OR ‘physical exercise*’[tiab] OR ‘sport*’[tiab]) AND (‘Neoadjuvant Therapy’[Mesh] OR ‘cancer treatment*’[tiab] OR ‘chemo’[tiab] OR ‘chemotherapeutic*’[tiab] OR ‘chemotherap*’[tiab] OR ‘cancer therapy*’[tiab]) limited to randomised controlled trial (RCT) and observational studies (OS), full text, English language, adult population, for preclinical and clinical studies published between January 1999 and February 2022. A hand search for unpublished trials and a review of reference lists was also performed. A second updated search was performed in September 2022.

### 2.2 Study Selection

Two reviewers (R.W., P.P.) screened titles and abstracts and removed papers according to inclusion and exclusion criteria, full texts of the remaining studies were assessed. ClinicalTrials.gov was also manually reviewed to check the trial status.

Inclusion criteria were clinical trials, OS and feasibility studies that investigated exercise as an intervention in adults > 18 years old undergoing chemotherapy, irrespective of tumour type, site, stage, patient sex and type of chemotherapy. Exercise programmes were included if given individually or as a group, supervised or unsupervised at home or not. Aerobic, anaerobic, resistance or flexibility exercises or combinations were all included. Exclusion criteria were dance, yoga and tai chi interventions and trials where the intervention occurred after oncological therapy had concluded.

### 2.3 Data Extraction

Data was extracted from each study and tabulated. When two studies duplicated results for the same trial, unique datapoints were included and where there was duplication or discrepancy the latter study data was included. Neoadjuvant chemotherapy, combination chemotherapy with curative intent and palliative chemotherapy regimes were included. Risk of bias was assessed and graded using the Cochrane Collaboration risk of bias tool [10]. Participant blinding was not considered due to the nature of the interventions.

## 3 Results

**Figure 1:**
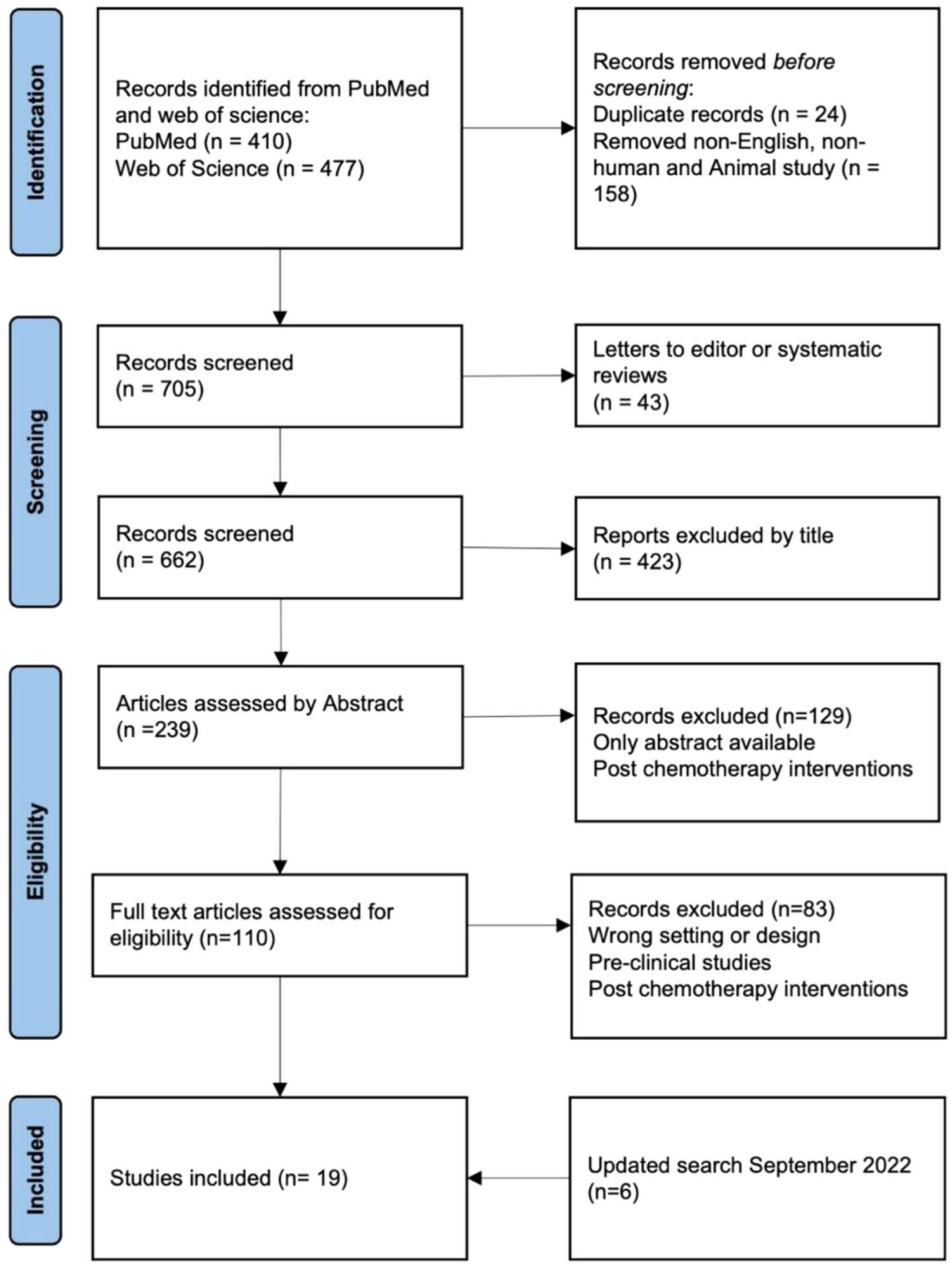
Prisma flow diagram, 705 records were screened by two reviewers and 19 studies included in the final review.

After full‐text analysis, 19 clinical studies (breast [*n* = 6], oesophagogastric [OG] [*n* = 8], head and neck [*n* = 1], colorectal [*n* = 3] and mixed cancer [*n* = 2]) (Figure 1) were included in the final analysis. Summarised trial characteristics for each study are included in Tables 1 and 2. The majority of studies (10/19 [53%]) included patients having chemotherapy in a neoadjuvant setting, although some studies also included patients undergoing palliative chemotherapy. Less than 50% of the trials were at low risk of bias for blinding the outcome assessor. All other aspects of risk of bias were low risk (Figure S1).

**Table 1:** Included studies and trials numbers of participants and exercise interventions.

Author	Study	Intent	No of participants			Location delivered	Duration	Exercise	Exercise type	Cancer type	Study design	Country
			Total	Intervention	Control			Sessions/week				
Randomised controlled trials												
Allen et al. [12]		Neoadjuvant	48	24	24	Hospital & home	15 weeks	2 × 60 min & 3 × 60 at home	A, R, F	OG	Randomised controlled trial	UK
Ariza‐Garcia et al. [4]	e‐CuidateChemo	Any	68	19	20	Home (web‐based)	8 weeks	3 × 35 min	A	Breast	Randomised controlled trial	Spain
Kleckner et al. [8]	EXCAP	Any	293	170	185	Home	6 weeks	3–5 × 20–60 min	A, R	Mixed	Randomised controlled trial	USA
Lee et al. [6]		Primary	30	15	15	Gym (at hospital)	8 weeks	3 × 30 min	A, HIIT	Breast	Randomised controlled trial	USA
Lin et al. [18]		Any ( > 6 month est survival)	40	20	20	Gym (at hospital)	8 weeks	3 × 90 min	A, R, F	Head & neck	Randomised controlled trial	Taiwan
Mijwel et al. [14]	OptiTrain	Adjuvant	240	74 (R) & 72 (A)	60	Gym (at hospital)	16 weeks	2 × 60 min	R HIIT & A HIIT	Breast	Randomised controlled trial	Sweden
Morielli et al. [22]	EXERT	Neoadjuvant	32	16	16	Gym (at hospital)	9 weeks	3 × 30 min	A	Colorectal	Randomised controlled trial	Canada
Müller et al. [5]	PIC	Any	142	43 (SMT) & 52 (R)	58	Gym (at hospital)	20 weeks	3 × 35 min AT or 2 × 45 min RT	SMT, R	Breast	Randomised controlled trial	Germany
Rao et al. [7]		Neoadjuvant	10	5	5	Home, supervised Bootcamp	4−6 months	3 × 60 min	A, R	Breast	Randomised controlled trial	USA
Stuecher et al. [17]		Any	28	13	15	Home	12 weeks	5 × 30 min or 3 × 50 min	A	OG	Randomised controlled trial	Germany
Moug et al. [15]	REx	Neoadjuvant	48	18	22	Home	14 weeks	Incremental increase in step count to 3000	A	Colorectal	Randomised controlled trial	UK
Non‐randomised studies												
Zylstra et al. [16]	Pre‐EMPT	Neoadjuvant	40	21	19	Home	20 weeks		A, R	OG	Non‐randomised controlled trial	UK
Leach et al. [20]	BEAUTY	Any	63	63	N/A	Home	12 weeks	2 × AT 1 × RT 5−7 × F 20–60 min	A, R, F	Breast	Observational study	Canada
Grabenbauer et al. [13]		Any	37	37	N/A	Gym (private)	12 weeks	2 × 30−60 min	A	Mixed	Observational study	Germany
Halliday et al. [19]	PREPARE	Neoadjuvant	79	51	28	Home	16 weeks	Personalised prescription	A, R	OG	Non‐randomised controlled trial	UK
Janssen et al. [21]	PREPARE	Neoadjuvant	95	52	43	Home	20 weeks	Personalised prescription	A, R	OG	Non‐randomised controlled trial	The Netherlands
Argudo et al. [23]		Neoadjuvant	33	33	N/A	Hospital	5 weeks	5 × 60 min	HIIT	OG	Feasibility study	Spain
Christensen et al. [11]		Neoadjuvant	50	21	29	Hospital	12 − 20 weeks	2 × 75 min	HIIT, R	OG	Non‐randomised controlled trial	Denmark
Chmelo et al. [35]	ChemoFit	Neoadjuvant	42	42	N/A	Home	91 days	Incremental increase in step count	A	OG	Feasibility study	UK

Abbreviations: A = aerobic, F = flexibility, HIIT = high intensity interval training, R = resistance, SMT = sensorimotor training.

**Table 2:** Trials and significant or frequently reported outcomes.

		Usual care			Exercise		*p*
Chemotherapy completion rate							
Allen et al. [12]	Completion rate	46%			75%		0.036
Müller et al. [5]	Achieved RDI 85%	76%			94%		0.032
Body composition/BMI							
Allen et al. [12]	Muscle mass %	−15.6%			−11.6%		0.049
Steucher et al. [17]	Lean body mass % change	0.64%			3.40%		0.02
Grabenbauer et al. [13]					0 months	3 months	
	BMI kg/m^2^				27.4	25.9	0.001
	Median fat mass %				30.70%	28.90%	0.001
Mijwel et al. [14]		0 months	12 months		0 months	12 months	
	BMI kg/m^2^	24.96	26.05	AT	24.64	24.17	< 0.001
				RT	25.38	24.55	< 0.021
Zylstra et al. [16]		0 months	Post‐chemo		0 months	Post‐chemo	
	Weight kg	87.5	88.2		80.1	76.4	0.05
	Fat free mass index %	16.3	14.7		17.8	18.7	0.026
Lin et al. [18]		0 months	2 months		0 months	2 months	
	Body fat %	25.9%	25.8%		25.5%	21.0%	0.002
Halliday et al. [19]			Post‐chemo			Post‐chemo	
	Relative skeletal muscle area		−10.6%			−6.1%	0.039
	Relative skeletal muscle index change %		−10.6%			−6.3%	0.05
Christensen et al. [11]					0 months	Post‐chemo	
	Lean body mass kg				55.6	57	NS
	Fat mass kg				29.6	32	NS
	Body fat %				33.6%	35.1%	NS
Moug et al. [15]		0 months	3 months		0 months	3 months	
	BMI kg/m^2^	28	28.1		26.5	26.8	
Chmelo et al. [35]					0 months	3 months	75% CI
	Lean body mass kg				52.3	49.1	−3.8; −2.5
	CT defined sarcopoenia present, *n* (%)				17 (47.2%)	26 (72.2%)	N/A
Strength and sarcopoenia							
Allen et al. [12]	Handgrip strength (kg)	−0.2			4.6		0.016
Mijwel et al. [14]		0 months	12 months		0 months	12 months	
	Handgrip strength (kg)	24.96	26.05	AT	28.44	29.93	< 0.001
				RT	28.4	29	< 0.021
Lin et al. [18]		0 months	2 months		0 months	2 months	
	Muscle mass %	31.5	31.4		34.1	34.5	0.008
	Upper limb strength (reps/30 s)	23.4	21.06		24.1	27	0.037
	Lower limb strength (reps/30 s)	15.6	13.1		19.7	20.14	0.025
Müller et al. [5]		0 months	Post‐chemo		0 months	Post‐chemo	
	Lower limb strength (nm)	148.5	134		160.9	164.2	< 0.001
Ariza‐Garcia et al. [4]		0 months	2 months		0 months	2 months	
	Abdominal strength (secs holding position)	48.6	30.01		29.01	53.94	< 0.001
	Back strength (kg)	39.27	40.66		41.05	53.5	< 0.001
	Lower limb strength (secs sit to stand)	23.23	24.5		24.3	21.47	< 0.05
	Handgrip strength (kg)	23.76	25.08		23.41	25.45	N/S
Christensen et al. [11]					0 months	2 months	Mean difference
	Leg press (kg)				116.4	143.9	26.9
	Knee extension (kg)				50.1	60.4	9.9
	Chest press (kg)				31.3	36.8	5.1
	Row (kg)				59.2	68.9	8.9
Chmelo et al. [35]					0 months	3 months	75% CI
	Handgrip strength (kg)				39.4	33.6	−2.6; 1.0
VO_2max_/fitness							
Allen et al. [12]	VO_2__max_ mL/kg/min	−2.5%			−0.4%		0.022
	Anaerobic threshold mL/kg/min	−6.70%			−3.70%		N/S
Leach et al. [20]					0 months	6 months	
	VO_2max_ mL/kg/min				27.9	29.8	< 0.05
	Duration of submax treadmill (min)				12.8	13.9	0.001
Grabenbauer et al. [13]					0 months	3 months	
	VO_2max_ mL/kg/min				18.8	20.5	0.005
	Median fat mass %				30.70%	28.90%	0.001
Ariza‐Garcia et al. [4]		0 months	2 months		0 months	2 months	
	6‐minute walk test (m)	480.13	453.79		421.83	483.46	< 0.05
Lee et al. [6]		0 months	2 months		0 months	2 months	
	6‐minute walk test (m)	436.82	430.23		439.8	490.8	0.008
	Margaria−Kalamen stair climb test (s)	4.66	4.98		3.84	3.71	0.013
Chmelo et al. [35]					0 months	3 months	
	VO_2max_ mL/kg/min				19.4	19.3	NS
	Anaerobic threshold mL/kg/min				14.3	13.9	NS

In total, 1418 patients were recruited across 19 trials, consisting of 11 (RCTs) and eight OS. Among these, five trials included only female participants with breast cancer. The representation of females was notably higher in breast cancer trials, while the opposite was observed in trials focusing on OG tumours, aligning with the demographic trends among patients affected by these conditions.

Ten studies had a supervised intervention, while eight trials required participants to record activity in a diary or by using an e‐watch un‐supervised. The interventions were delivered at facilities such as a hospital (*n* = 3), gym at a healthcare facility (*n* = 5), a private gym (*n* = 1), individual home (*n* = 9), a combination of both home and facility (*n* = 2) and patient choice of either location home or healthcare facility (*n* = 2). One trial conducted an online supervised programme at home [4] and one trial recruited patients to a ‘bootcamp’ [7]. In all 19 studies, there were no adverse events attributable to exercise.

### 3.1 Intervention

Six studies implemented only aerobic training (AT) such as walking, cycling, jogging and inclined treadmill; four trials used both AT and resistance training (RT), three trials implemented a combination of AT, RT and flexibility training (FT), one trial compared RT to sensorimotor training and a further trial compared AT to RT and usual care (UC). High intensity interval training (HIIT) was used in four studies and in one study in combination with RT.

The exercise programme in all trials, including the training type, intensity and session duration, were adjusted according to the patient's ability and motivation.

All 11 RCTs and four of the OS had a UC control group, which received no exercise prescription. In one trial, participants in the UC group were asked not to exceed a total of 30 min of physical activity per week [6]. For the remaining UC groups, verbal or written information about physical activity was given at the initial consultation. In all trials, the UC group received contact from study coordinators to ensure the same social and nutritional support.

The duration of intervention varied between trials, ranging from 6 weeks to more than 26 weeks. In some trials, the intervention period was fixed, whereas in other trials, the intervention period was linked to a variable duration of chemotherapy. A summary of the intensity, frequency and the duration of exercise sessions can be found in Table 1. The heterogeneous interventions reflects the uncertainty in the literature as to what exercise intervention delivers the most effective benefits.

#### 3.1.1 Chemotherapy Completion Rate

No two studies used the same metric to define completion rate. A study of exercise during NAC in OG cancer [11] reported a completion rate of 95% in the exercise group compared to 79% in the UC arm (significance level not reported). They defined failure to complete treatment as any cancelled treatment including surgery. However, this observational study included more smokers in the control arm, potentially confounding results.

Also in OG cancer, one RCT [12] in 54 patients (26 exercise, 28 UC) compared exercise (AT, RT and FT) and psychological support with UC. Seventy‐five percent of participants in the exercise group completed all planned cycles of NAC, compared to 46% in the control group (*p* = 0.036). The PIC trial [5] recruited patients undergoing chemotherapy for any cancer, they randomised 170 patients to either RT, SMT or UC. Ninety‐four percent of patients reached a clinically relevant relative dose intensity (RDI) threshold of 85% in the exercise groups, compared to 76% in the UC group (*p* = 0.032).

#### 3.1.2 Body Mass Index (BMI) and Body Composition

There was no universally applied measure of optimising body composition. BMI was reported by three trials. In a home AT programme in 45 patients undergoing cancer treatment Grabenbauer et al. [13] reported that median BMI decreased from 27.4 to 25.9 kg/m^2^ at 3 months (*p* = 0.001). The OptiTrain trial [14] compared 16 weeks of HIIT, combined with either AT or RT, to UC during chemotherapy in 240 women. BMI was reduced in both the AT (24.64 vs. 24.17 kg/m^2^, *p* < 0.001) and RT (25.38 vs. 24.55 kg/m^2^, *p* < 0.021) groups compared to UC at 12 months. Conversely the REx trial in colorectal cancer [15] demonstrated no change in BMI between groups in a home based, step‐count measured, trial.

Total weight was reported by Zylstra et al. [16] In a prospective trial of 40 patients (21 AT, 19 UC) undergoing NAC for oesophageal cancer, weight remained stable in the AT group compared to a weight gain in the UC group (−0.5% vs. 1.2%, *p* = 0.05). Zylstra et al. [16] also report fat free mass index was improved in the AT group (AT 17.8 vs. 18.7 kg/m^2^; UC 16.3 vs. 14.7 kg/m^2^, *p* = 0.026). Lean body mass also improved in the AT group in Steucher et al.'s [17] randomised 12 week trial in patients undergoing chemotherapy for gastrointestinal cancer (+3.4% vs. +0.64%, *p* = 0.02). Lin et al. [18] in 40 patients (20 exercise, 20 UC) undergoing chemotherapy for head and neck cancer reported a reduction in mean body fat percentage in the exercise arm (exercise 25.5% vs. 21%; UC 25.9% vs. 25.8%, *p* = 0.002). Body fat percentage decreased from 30.7% to 28.9% at 3 months (*p* = 0.001) with exercise in Grabenbauer et al.'s [13] study. However in OG cancer Christensen et al. [11] reported no significant changes in lean body mass, fat mass or fat percentage in patients with HIIT and RT.

#### 3.1.3 Skeletal Muscle Strength and Sarcopenia

Halliday et al. [19] measured skeletal muscle area (SMA) at midpoint of the third lumbar vertebra and skeletal muscle index (SMA:height^2^) both measures fell, but by less in the intervention group, SMA (−6.1% vs. −10.6%; *p* = 0.039) and skeletal muscle index (−6.3% vs. −10.6%; *p* = 0.05).

Lin et al. [18] demonstrated increases in skeletal muscle mass (intervention 34.1% vs. 34.5%; control 31.5% vs. 31.4%, *p* = 0.008). Whereas Allen et al. [12] reported less muscle mass loss (intervention −11.6 vs. −15.6 controls, cm^2^/m^2^; *p* = 0.049).

Handgrip strength increased in the Optitrain study (+ 3.23 kg, *p* < 0.001) [14] and, in patients without sarcopoenia at baseline, hand grip strength also improved in Allen et al.'s [12] intervention group (+4.6 vs. −0.2 kg controls; *p* = 0.016).

Lin et al. [18] demonstrated an increase in upper (intervention 24.1 vs. 27.0 reps/30 s; control 23.4 vs. 21.06 reps/30 s, *p* = 0.037) and lower limb strength (intervention 19.7 vs. 20.14 reps/30 s, control 15.6 vs. 13.1 reps/30 s, *p* = 0.025).

In the e‐CuidateChemo study [4], abdominal strength (24.93 vs. −18.59 s [seconds holding positions; longer = better]), back strength (12.45 vs. 1.39 kg [lumbar resistance]) and lower body strength (−2.82 vs. 1.26 s, [sit to stand test; shorter = better]) all improved in exercise groups (all *p* < 0.001).

Similarly, the PIC trial [5] reported improved quadriceps muscular strength in patients who were compliant with the intervention (intervention 160.9 vs. 164.2; control 148.5 vs. 134, *p* < 0.001).

#### 3.1.4 VO_2__max_/Physical Fitness

In Christensen et al. [11], there was no fall in VO_2__max_ in exercised individuals with oesophageal cancer (25.23 vs. 26.62). Allen et al. [12] demonstrated that decreases in VO_2__max_ could be attenuated in exercised groups versus controls (mean change in the exercise group −0.4 [95% CI −0.8 to 0.1] vs. controls −2.5 [95% CI −2.8 to −2.2] mL/kg/min; *p* = 0.022) but no effect was seen on the trial's primary end point: anaerobic threshold. Grabenbauer et al. [13] demonstrated VO_2__max_ increased from 18.8 to 20.5 mL/min/kg at 3 months (*p* = 0.005) and 20.0 mL/min/kg at 12 months (*p* = 0.003). Likewise, the BEAUTY study [20] demonstrated improved VO_2__max_ in exercised individuals at 24 weeks (+ 1.9 mL/kg/min, *p* = 0.018) and an increase of approximately 1 min in the submaximal treadmill test. (*p* = 0.013).

Janssen et al. [21], however, measured a fall in VO_2__max_ in exercised individuals and not in the control group.

Lee et al. [6] randomised patients with breast cancer to either AT HIIT (*n* = 15) or UC (*n* = 15). Improvements were found for the Margaria−Kalamen stair climb test (−0.13 vs. +0.32 s, *p* = 0.013) and 6 minute walked test (6MWT) (+51 vs. −6.59 m, *p* = 0.008). 6MWT also improved in the e‐CuidateChemo [4] study (+ 15.42 m, *p* = 0.015).

In the Lee et al. [6] trial, a composite physical fitness index fell in the control group but was unchanged in the intervention group (intervention 56.7 vs. 64.7, *p* = 0.237; control 78.0 vs. 67.6, *p* = 0.031) and heart rate recovery was worse in the control arm after chemotherapy whereas it was unchanged in the intervention group (HR/2 at 3 min; intervention 44.4 vs. 41.9 *p* = 0.237; control 33.7 vs. 40.6, *p* = 0.003). This was despite better heart rate recovery in the control arm versus the intervention arm before chemotherapy (HR/2 at 3 min; intervention 44.4, control 33.7, *p* = 0.005).

#### 3.1.5 QoL and Global Health Status

##### 3.1.5.1 Cognitive Function

Lin et al. [18], the BEAUTY trial [20] and the PIC study [5] found no significant changes in cognitive function using the EORTC QL‐C30 questionnaire. In the EXERT trial [22], however, cognitive function was worse in the exercise group (EORTC QL‐C30, intervention 82.3 vs. 79.2; control 84.4 vs. 91.7, *p* = 0.028).

##### 3.1.5.2 Global Health Status

Ten trials assessed QoL; six of these trials have reported a significant improvement in QoL and global health status with exercise. Allen et al. reported improved global QoL in the 6 weeks post‐surgery (EORTC QL‐C30 global health score: Intervention 83.8; control 59.06, *p* = 0.001) [12].

Lin et al. [18] and Müller et al. [5], using the EORTC QL‐C30 questionnaire, both revealed significant improvements in the exercise group. Lin et al.: (EORTC QL‐C30 intervention 58.5 vs. 71.0; control 57.5 vs. 54.5, *p* = 0.001) and the PIC trial (EORTC QL‐C30 intervention 60.0 vs. 69.3; control 62.7 vs. 56.4, *p* = 0.005).

Grabenbauer et al. [13] and Argudo et al. [23] reported improvement in global health scores over duration of treatment (*p* < 0.001) but they were without control arms.

In the BEAUTY trial [20], The Functional Assessment of Cancer Therapy‐Breast (FACT‐B) scores improved at 24 weeks compared to both baseline and 12 weeks (*p *= 0.002 and 0.001).

#### 3.1.6 Biomarkers

##### 3.1.6.1 Inflammation

Two trials provided data on inflammatory biomarkers. The EXCAP trail [8] compared concentrations of pro‐inflammatory and anti‐inflammatory cytokines. Pro‐inflammatory markers decreased significantly through exercise (IFN‐γ, *p* = 0.030; IL‐8, *p* = 0.005; and IL‐1β, *p* < 0.0001), whereas only one pro‐inflammatory marker (IL‐8) decreased significantly in controls (IFN‐γ, *p* = 0.813; IL‐8, *p* = 0.005; IL1β, *p* = 0.073). Comparing exercise and control arms only IFN‐γ displayed a significant reduction in concentration (*p* = 0.044).

For anti‐inflammatory markers, all three increased significantly in the exercise group (IL‐6, *p* = 0.020; IL‐10, *p* = 0.0004; and sTNFR1, *p* < 0.0001), whereas only two (IL‐10 and sTNFR1) increased significantly in controls (IL‐6, *p* = 0.395; IL‐10, *p* < 0.0001; and sTNFR1, *p* < 0.0001). There was no statistically significant difference between groups.

The PRE‐EMPT trial [16] demonstrated significant changes following NAC. IL‐6 concentrations significantly increased in both exercise and control arms (*p* = 0.044) but to a greater extent in controls (% change, exercise 27.93 vs. 126.41 control, *p* = 0.04). Increases in TNFa and IFN‐γ were not significant (*p* = 0.25, *p* = 0.36) in either group. Cytotoxic T lymphocyte levels were significantly higher in exercise (CD3+ % change, exercise 34.26 vs. 4.53 control, *p* = 0.03; CD8+ % change, exercise 29.41 vs. 0.98 control, *p* = 0.03). A small pilot study investigated the change in KI‐67 expression on tumour cells, the results were not significant [7].

#### 3.1.7 Tumour and Lymph Node Regression

Zylstra et al. [16] compared response to chemotherapy using the Mandard tumour regression grading (TRG) in the primary tumour and lymph nodes. More tumours were defined as responders in the exercise than in the control arm (responders [Mandard TRG1‐2], exercise 7/21 vs. 1/19 control, *p* = 0.044). Results of tumour regression in the lymph nodes trended towards improvement in the exercise arm but did not reach significance (*p* = 0.077).

## 4 Discussion

There is little doubt that exercise improves overall health. However, the surgical and oncological communities have been reticent to embrace exercise as a standard part of cancer therapy, partly due to the financial and infrastructural barriers to delivering exercise programmes. Exercise improves strength, fitness, fat free mass, BMI and QoL [14, 16, 18, 20] and the beneficial effects are achievable during cytotoxic anticancer therapy. In 19 studies, there were no reported adverse events attributable to exercise. Exercise improves tolerance and compliance with chemotherapy regimens and this alongside immunological/inflammatory factors may improve the anticancer effects of chemotherapy [8, 16]. This may have implications for disease free and overall survival and future trials should investigate this.

In this systematic review, we investigated the effect of exercise on a range of outcomes during chemotherapy. The mode of exercise varied in each trial, and included aerobic, resistance, flexibility, HIIT or combinations thereof, supervised at home, at a facility or unsupervised. There was significant variation in the nomenclature and terminology used to describe interventions both conceptually and specifically. A plethora of outcomes were recorded in individual trials with the majority being surrogate markers for a clinically meaningful effect.

Different cancer types have varying treatment algorithms and certain malignancies may be better suited to prehabilitation due to the nature of the treatment pathway. Gastrointestinal cancers such as gastro‐oesophageal and rectal cancer are frequently treated with neoadjuvant therapy [24, 25] before a surgical intervention. As a result they are an ideal patient cohort for a prehabilitation programme as a window of opportunity exists in their treatment pathway to rescue a decline in physical fitness due to neoadjuvant treatment [26, 27]. Whilst most prehabilitation studies have historically focussed on surgical patients, often targeting an intervention within a very narrow time window before surgery, it is important that patients in other settings (e.g., palliative) are not neglected. The Macmillan guidelines clearly state that prehabilitation should be considered for all patients (34) and many of the benefits discussed in this review are not exclusive to surgical patients. Clearly, there are significant resource implications for expanding eligibility criteria for prehabilitation to all patients. However, in the context of other costs incurred in the field of oncology, for example, drug expenditure, exercise remains a very cost‐effective intervention.

Established guidelines that healthcare providers can follow to prescribe a tailored exercise programme are lacking. No trial was able to show the benefit of one exercise intervention over another and this area requires more research. Future studies should investigate the optimum exercise forms and intensities. In this regard, there is a conundrum. An ideal exercise trial would have a homogenous intervention facilitating a robust scientific comparison of intervention and control groups. In reality, patients have a wide variety of co‐morbidities, physical limitations and exercise interests. A tailored exercise programme, as advocated by a number of guideline documents (e.g., Macmillan), is most likely to improve patient engagement and compliance. However, this tailored approach introduces heterogeneity into trial interventions. The only way around this is for high quality studies to interrogate specific aspects of the intervention to inform prehabilitation programmes moving forward whilst at the same time acknowledging that exercise is now the standard of care in cancer treatment. As such, not all patients need to be enroled in idealistic experimental trials to gain the aforementioned benefits and this more pragmatic approach may improve rates of adoption.

We identified eight trials currently running worldwide investigating the effect of exercise during chemotherapy. These include further disparate measurements of the effect of exercise, such as muscle biopsies, body mass composition, with a more expanded study on inflammation, inflammatory markers and biomarkers of cardiovascular function, as well as vascular stiffness.

## 5 Limitations and Future Research

The studies are heterogeneous with small sample sizes. Most studies focussed on subjective QoL measures or surrogate markers of health. It is unclear what a ‘good outcome’ represents when exercise is the intervention. Cancer is a catabolic disease and weight maintenance can be a challenge to allied health professionals and patients. Conversely, obesity is a risk factor for many cancers and a risk factor for operative complexity and complications. In broadly discussing ‘BMI’ our aims are necessarily more nuanced, and in some individuals, weight maintenance would represent a clinically meaningful result, whereas, in others, substantial weight loss may be the desired outcome. Interpreting the value of BMI as an outcome measure per se is therefore challenging. Moreover the interaction between loss of fat free mass versus loss of skeletal muscle function and changes in physical fitness in a perioperative cancer context needs to be interrogated.

Sarcopenia has been shown to influence outcomes in OG and other cancers [28, 29] and sarcopenia can be ameliorated by exercise [16, 17]. However, no trial to our knowledge has yet shown that preventing or reversing sarcopenia improves patient outcomes. Moreover, sarcopenia may not be the best objective preoperative predictor of poor outcome and CPET variables such as VO_2__max_ and anaerobic threshold may be better [30, 31]. While VO_2__max_ and anaerobic threshold can be improved with exercise, the subsequent effect on survival remains unknown.

A consensus core outcome set (COS) for prehabilitation is lacking. In designing future trials, the views of patients and experts need to be included to inform a COS. Patient involvement in designing a COS will lead to better trial design and more patient relevant primary and secondary endpoints. Despite the evidence for the benefits of exercise, that may translate into long term survival advantages, no trial has reported on long term disease free or overall survival.

Currently published studies are limited by sample size yet, despite these low numbers, differences in selected endpoints reached significance. Taken together, the clear implication is of a range of improvements in exercising patients undergoing chemotherapy although numerous questions around the nature, location and monitoring of exercise interventions persist. Higher powered trials, with longer follow‐ups are required, both to confirm these findings and explore the mechanisms behind these effects.

The modern concept of prehabilitation incorporates at least three components—exercise, nutrition and psychology [32]. The latter two are often neglected as evidenced by a lack of these interventions presented in this review. This is despite the significant nutritional issues experienced, particularly by patients undergoing major gastrointestinal surgery, and the psychological morbidity of a cancer diagnosis and its subsequent treatment. Existing studies investigating nutritional interventions during cancer treatment are hampered by the same issues we found with exercise. Namely heterogeneity in baseline measurements, interventions and primary and secondary endpoints [33]. The European Society for Clinical Nutrition and Metabolism, ESPEN, have published guidelines on nutrition in cancer patients [34] but a COS to address the issues of heterogeneity is lacking.

Few studies have reported on the ethnicity of the participants and none on the barriers to participation in exercise programmes, which may be considerable. In studies that included socioeconomic status, most participants were White with a high school or higher education which may reflect the demographics of the tumour groups in question or a potential selection bias for entry into exercise studies.

To date, only one study has investigated outcomes in patients who declined participation in or dropped out of prehabilitation programmes. This study indicates that the survival rate worsens for these patients. All of these factors may represent missed cohorts of participants who, for a variety of reasons, may be at greatest need for interventions that improve health outcomes.

## 6 Conclusions

This systematic review demonstrates an overall positive effect of exercise on outcomes during chemotherapy. Markers of body composition, fitness and QoL were all improved by the introduction of an exercise programme. In addition, there is evidence of improved anti‐inflammatory and immune responses with exercise and potentially improved response to chemotherapy. Together, these findings suggest that exercise during chemotherapy can improve health, fitness, QoL and potentially the effectiveness of chemotherapy which may translate into improved long‐term outcomes. Before large adequately powered trials are launched, a COS of validated clinical parameters should be developed. Exercise, nutritional support and psychological input form the three component parts of the prehabilitation concept. Whilst a greater understanding of the optimal intervention is clearly required, this should not prevent prehabilitation from now being considered a standard of care in patients being treated for cancer in all settings.

## Conflicts of Interest

The authors declare no conflicts of interest.

## Synopsis

A systematic review evaluated the effect of exercise prehabilitation on patients undergoing chemotherapy. Nineteen studies were analysed involving 1418 patients. Results suggest that exercise interventions may enhance body composition, fitness, strength and quality of life.

## Supporting information

Supporting information.

## Data Availability

Data sharing is not applicable to this article as no new data sets were generated or analysed in this study.
