# Supplementary material for: Exercise During Chemotherapy for Cancer: A Systematic Review
Source: J Surg Oncol. 2024 Oct 23;130(8):1725–36. doi: 10.1002/jso.27845 (PMC11849706; doi:10.1002/jso.27845)
Supplement: Supplementary file 1 — Supporting information. [file JSO-130-1725-s001.pdf]

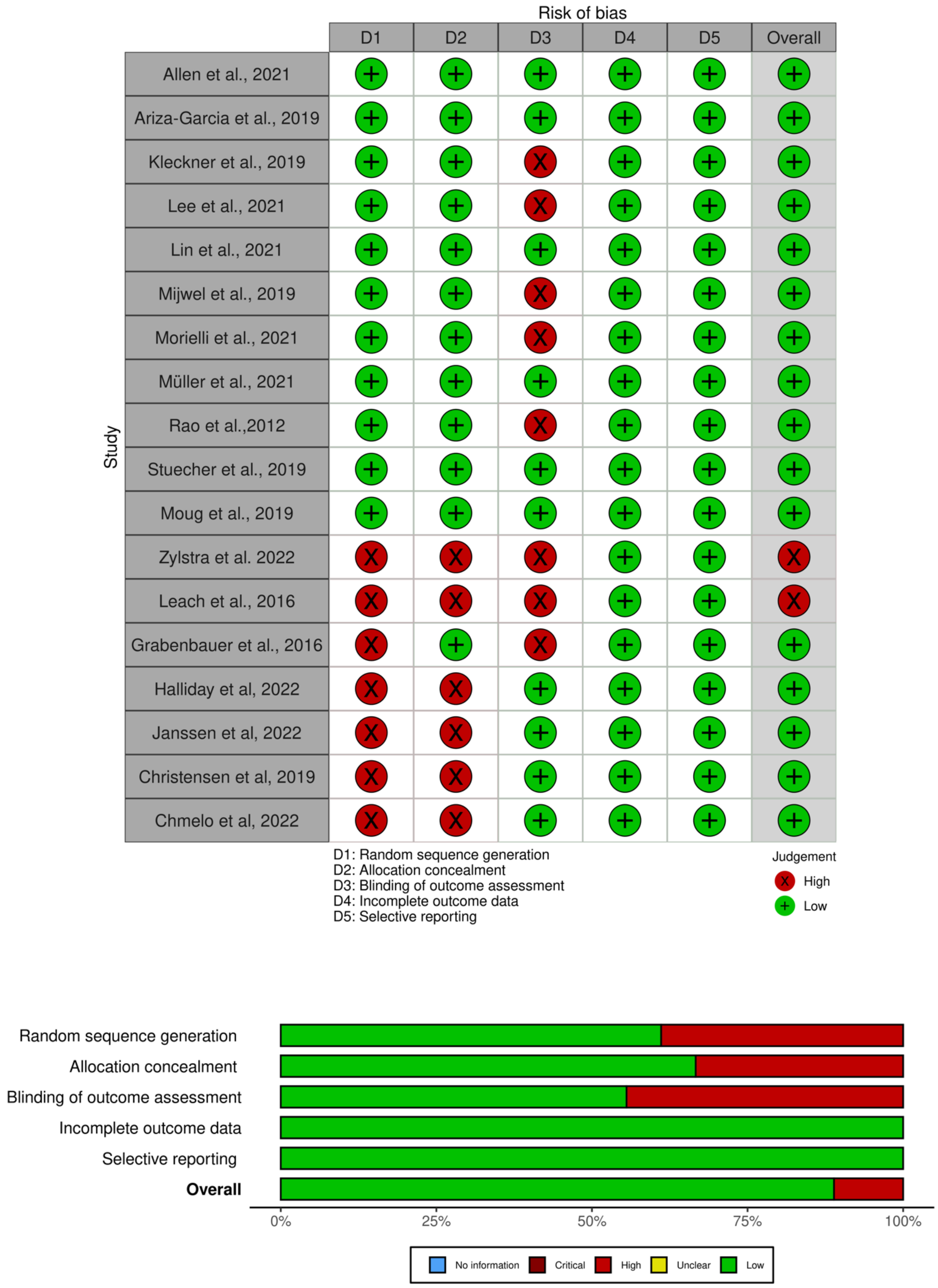

Supplementary Figure 1 Risk of bias assessment.
